# Potential Distribution and Response to Climate Change in *Puccinellia tenuiflora* in China Projected Using Optimized MaxEnt Model

**DOI:** 10.3390/biology14101426

**Published:** 2025-10-16

**Authors:** Hao Yang, Xiaoting Wei, Manyin Zhang, Jinxin Zhang

**Affiliations:** 1 Institute of Ecological Conservation and Restoration, Chinese Academy of Forestry, Beijing 100091, China; yanghao_0618@163.com (H.Y.); weixiaoting6@163.com (X.W.);; 2 Grassland Research Center, National Forestry and Grassland Administration, Beijing 100091, China

**Keywords:** climate change, human activities, MaxEnt model, potential suitable habitat, *Puccinellia tenuiflora*

## Abstract

**Simple Summary:**

Climate change and human activities are increasingly threatening grassland ecosystems, especially salt-tolerant plants that play key roles in ecological restoration. This study focuses on *Puccinellia tenuiflora*, a hardy grass widely used to improve saline–alkali soils in northern China. Using an optimized MaxEnt model, we project that over half of the current suitable habitat for this grass may disappear by the 2090s under high emissions, with habitats shifting northeast. Human activities and temperature variations are primary drivers. These findings highlight the potential vulnerability of this important species to future climate change and human activities and provide a scientific basis for its conservation and sustainable use in vegetation restoration projects.

**Abstract:**

Global climate change is accelerating and human pressures are intensifying, exerting profound impacts on biodiversity and ecosystem service functions. The accurate prediction of species distributions has thus become a critical research direction in ecological conservation and restoration. This study selected *Puccinellia tenuiflora*, a species distributed across China, as its research subject. Utilizing 169 occurrence records and 10 environmental variables, we applied a parameter-optimized MaxEnt model to simulate the species’ current and future (2050s–2090s) potential suitable habitats under the SSP126, SSP370, and SSP585 scenarios. The results identified the human footprint index (HFI, 43.3%) and temperature seasonality (Bio4, 26.9%) as the dominant factors influencing its distribution. The current suitable area is primarily concentrated in northern China, covering approximately 258.26 × 10^4^ km^2^. Under all future scenarios, a contraction of suitable habitat is projected, with the most significant reduction observed under SSP585 by the 2090s (a decrease of 56.2%). The distribution centroid is projected to shift northeastward by up to 145.36 km. This study elucidates the response mechanism of *P. tenuiflora* distribution to climate change and human activities. The projected habitat contraction and spatial displacement highlight the potential vulnerability of this species to future climate change. These findings, derived from a rigorously optimized and spatially validated model, provide a scientific basis for the conservation, reintroduction, and adaptive management of *P. tenuiflora* under climate change.

## 1. Introduction

Grassland ecosystems, particularly the saline–alkali grasslands of northern China, are acutely vulnerable to the compounded effects of climate change and human activities, as highlighted in recent global assessments [1,2]. These ecosystems face significant degradation, threatening their critical role in soil conservation, forage production, and the maintenance of halophytic biodiversity [3,4]. Accurate prediction of the distribution of key halophytic species, such as *P. tenuiflora*, is therefore imperative for guiding effective restoration and conservation efforts in these sensitive and expanding habitats. Against this background, accurate prediction of species spatial distribution patterns and their response mechanisms to environmental change has become a critical issue in the fields of ecology, conservation biology, and global change research [5,6,7]. Species Distribution Models (SDMs) are pivotal tools for quantifying species’ niche requirements and predicting their potential habitats by establishing relationships between occurrence records and environmental variables [8,9,10]. The Maximum Entropy (MaxEnt) model was selected for this study due to its demonstrated robustness with presence-only data and small sample sizes, a common scenario for many plant species including *Puccinellia tenuiflora* (Turcz.) Scribn. et Merr. [11,12]. It is important to note that while MaxEnt remains widely applied, recent comparative studies have shown that ensemble modeling platforms (e.g., BIOMOD2) can provide superior performance, particularly when extrapolating to novel climatic conditions [13]. Furthermore, a key limitation of presence-only data is the inability to distinguish true absence from lack of survey effort. 

*P. tenuiflora*, a perennial halophyte of the grass genus Puccinellia, is recognized as a dominant and constructive species in saline-alkali regions of northern China [14,15]. Although not yet assessed on the IUCN Red List, it is identified as a key species for the restoration of degraded saline meadows in regional conservation strategies. Its ecological urgency is underscored by the rapid degradation of its native habitat; for instance, statistics indicate that over 90% of grasslands suffer from degradation in northern China due to agricultural reclamation and overgrazing [16,17]. This significant habitat loss threatens not only *P. tenuiflora* itself but also the critical ecosystem services it supports, making the accurate prediction of its suitable habitat a prerequisite for effective conservation planning and ecological restoration. In recent years, with the continuous expansion of salinized land area and growing demand for ecological rehabilitation, the species has been increasingly valued for its application in vegetation reconstruction and ecological engineering [18,19]. A systematic literature search conducted in May 2025 across Web of Science, Google Scholar, and CNKI using key terms related to *P. tenuiflora* and distribution modeling confirmed that while its physiological ecology [14,15,20] and local-scale patterns [21] have been studied, a comprehensive, national-scale prediction of its potential distribution integrating multi-dimensional environmental variables is currently absent. Previous research has predominantly focused on physiological and ecological aspects, such as salt-tolerance mechanisms and water-use efficiency [22,23], whereas macro-scale spatial prediction studies remain relatively limited and are often hampered by methodological constraints. Therefore, this study aims to fill this critical gap by providing the first systematic, nationwide projection of suitable habitats for *P. tenuiflora* under both current and future climate scenarios. We utilize a parameter-optimized modeling framework that explicitly addresses these methodological limitations through comprehensive variable selection and parameter tuning.

While this study focuses specifically on *P. tenuiflora* due to its paramount ecological role and the identified research gap, the optimized modeling framework (including variable selection, parameter optimization, and spatial validation protocols) established here is explicitly designed to be transferable. It provides a robust foundation for future comparative studies that aim to project the distributions of other congeneric species, such as *P. chinampoensis* and *P. distans*, and to uncover the mechanistic drivers of their varying responses to climate change. Analysis of the broader Species Distribution Model (SDM) literature reveals that the gap in *P. tenuiflora* research reflects persistent methodological limitations in macro-scale plant distribution modeling. Although incorporating non-climatic variables such as human footprint data represents an emerging trend [8], numerous studies remain predominantly reliant on climatic predictors, frequently overlooking the integrated effects of topography, vegetation, and human disturbance. Moreover, the widespread use of default model parameters without systematic optimization undermines predictive robustness and limits practical utility for precision ecological management under future climate scenarios [24,25,26,27]. In response, our study not only addresses the species-specific knowledge gap but also advances SDM methodology by (1) systematically integrating multi-dimensional environmental variables—climate, topography, NDVI, and HFI—and (2) implementing a rigorously parameter-optimized MaxEnt framework to enhance projection reliability for both current and future habitats.

There is currently no systematic research specifically targeting the spatial distribution prediction of *P. tenuiflora* conducted in China. This research gap prevents a rigorous assessment of the potential impacts of climate change on this species. Although studies on related species (e.g., *Stipa breviflora*) can provide partial methodological insights [28], obvious limitations remain in existing SDM research: Firstly, most models over-rely on climatic variables, while the combined effects of non-climatic factors such as topographic features, vegetation cover, and human disturbance are often neglected. Secondly, most existing studies directly adopt default model parameters, lacking systematic parameter optimization and validation procedures; this undermines the robustness of model predictions. More importantly, predictive studies on grass species distribution have largely focused on static simulations under current climatic conditions [24,25,26,27], with significant inadequacies in projecting dynamic changes in suitable habitats under future climate change scenarios.

Based on the identified research gaps, we propose the following three core hypotheses: H1: Integrating multi-dimensional environmental variables will yield a statistically superior model compared to a climate-only baseline, as determined by a ΔAUC > 0.02 and a ΔAICc > 2. H2: The Human Footprint Index (HFI) and temperature seasonality (Bio4) will be the dominant factors governing the distribution of *P. tenuiflora*, collectively contributing >85% to the total, with their response curves exhibiting significant non-linearities. H3: From the present to the 2090s, the total suitable habitat area will undergo a significant contraction, with the proportional habitat loss under SSP585 being at least 20% greater than under SSP126, and the distribution centroid will shift northeastward, with a displacement under SSP585 being 1.5 times greater than under SSP126. To test these hypotheses is crucial for reliably forecasting the species’ distribution dynamics and identifying the primary drivers of its range shifts. This study aims to achieve three primary objectives: (1) Compile a comprehensive dataset of species occurrences and multi-dimensional environmental variables for *P. tenuiflora* across China. (2) Develop an optimized species distribution model through systematic parameter tuning and variable selection. (3) Project the spatiotemporal dynamics of suitable habitats under current and future climate scenarios and identify key regions of habitat stability, loss, and gain. In summary, this study aims to clarify the potential distribution patterns of *P. tenuiflora* under different climate scenarios, thereby providing a scientific basis for the development of conservation, reintroduction, and adaptive management strategies for this species.

## 2. Materials and Methods

### 2.1. Occurrence Data Acquisition and Processing

**Figure 1:**
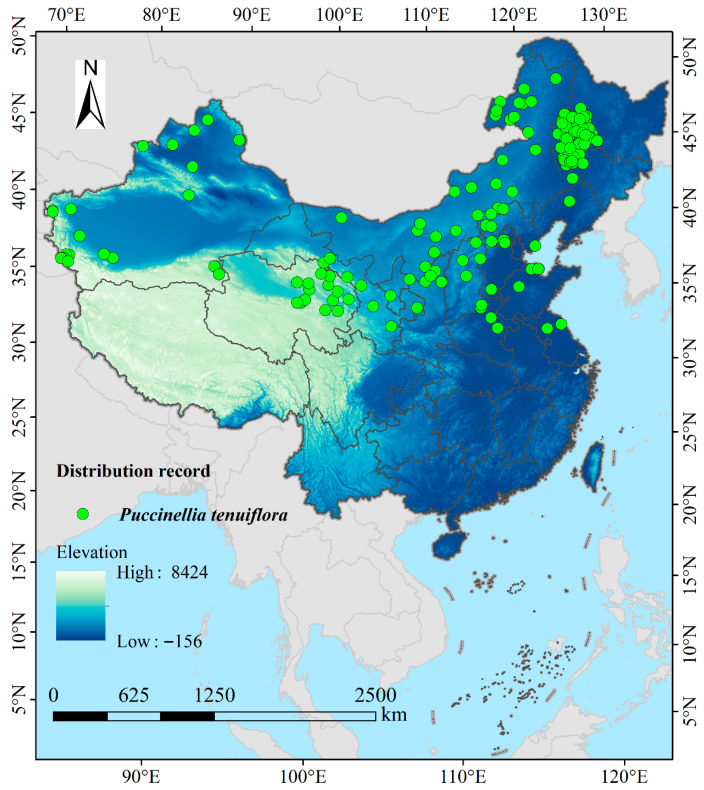
The geographical distribution records of *P. tenuiflora* in China.

The geographical distribution data of *P. tenuiflora* in China were primarily obtained from publicly available literature and specimen records through multiple sources, including the Chinese Virtual Herbarium (https://www.cvh.ac.cn/, accessed 26 May 2025), the Global Biodiversity Information Facility (GBIF Occurrence Download https://doi.org/10.15468/dl.z4esps, accessed 26 May 2025), and the National Specimen Information Infrastructure (http://www.nsii.org.cn/2017/home.php, accessed 27 May 2025). Additionally, data were sourced from the China National Knowledge Infrastructure (https://www.cnki.net/, accessed 27 May 2025), Web of Science (https://www.webofscience.com, accessed 27 May 2025), and Google Scholar (https://ac.scmor.com/, accessed 27 May 2025). For records that lacked explicit latitude and longitude coordinates but contained detailed locality descriptions at the town or village level, geographic coordinates were extracted using the online tool “Jingweidu Query Positioning” (http://jingweidu.757dy.com/, accessed 28 May 2025). For records with manually extracted coordinates, we quantified the potential georeferencing uncertainty by creating a 5 km radius buffer around each point, corresponding to the approximate spatial extent of town-level locality descriptions. The potential impact of this uncertainty was assessed by comparing model outputs with and without these buffered records in a sensitivity analysis. Subsequently, duplicate records with identical coordinates were removed using the “Remove Duplicates” function in Excel 2016. To reduce the effects of sampling bias and spatial autocorrelation on model predictions, the occurrence points were further processed using ArcGIS 10.8. Specifically, a spatial filtering method was applied at a resolution of 2.5 arc-minutes, whereby only the point closest to the center of each grid cell was retained [29,30]. The spatial resolution of 2.5 arc-minutes was chosen to match the finest resolution available for our key environmental variables (e.g., WorldClim bioclimatic layers). While this resolution may not capture micro-habitat heterogeneity, it is appropriate for a macro-scale national assessment and effectively captures the broad climatic gradients determining species distributions at this scope. To further mitigate spatial autocorrelation and sampling bias, we applied spatial thinning using the spThin R package (v0.2.0), retaining only one occurrence record per 5 km grid cell. This resulted in a final dataset of 169 spatially independent occurrences (Figure 1). Additionally, we incorporated a bias file in MaxEnt to weight background sampling probability based on the density of botanical collections across China, thereby reducing the influence of uneven sampling effort. Finally, the data were formatted as a CSV file according to the requirements of MaxEnt 3.4.4 (http://biodiversityinformatics.amnh.org/open_source/maxent/, accessed 8 June 2025).

### 2.2. Acquisition and Processing of Environmental Variables

A total of 24 environmental variables (Table S1) were systematically selected to comprehensively evaluate the effects of climate, topography, vegetation, and human activities on the distribution of *P. tenuiflora*. Specifically, 19 bioclimatic variables and elevation data were obtained from the WorldClim 2.1 global climate database (https://www.worldclim.org/, accessed on 22 May 2025). Based on the elevation data, slope and aspect were subsequently derived as topographic factors using ArcGIS 10.8. Furthermore, the intensity of human activity was represented by the Human Footprint Index [31], while vegetation coverage was quantified using the Normalized Difference Vegetation Index (NDVI) from the MOD13A3 product of the MODIS/Terra satellite [32]. To project the potential impacts of future climate change on the distribution of *P. tenuiflora*, the BCC-CSM2-MR climate model was employed, as it has demonstrated satisfactory performance in simulating climate patterns across China [33]. In this study, three Shared Socioeconomic Pathways (SSPs), namely SSP126, SSP370, and SSP585, were selected, representing low (~1.8 °C warming by 2100), medium (~2.7 °C), and high (~4.4 °C) emission scenarios, respectively [34]. Finally, the time periods considered included the 2050s (2041–2060), 2070s (2061–2080), and 2090s (2081–2100).

To mitigate potential interference from multicollinearity on model prediction accuracy and variable effect estimation, a comprehensive collinearity analysis was conducted. Specifically, Spearman correlation analysis and variance inflation factor (VIF) tests were performed for all environmental variables using the usdm R package (version 4.2.2). The screening criteria were a correlation coefficient of |r| < 0.7, to ensure stricter variable independence following recommendations for ecological data [35], along with a VIF value < 10, thereby ensuring the retention of variables with strong statistical independence [36]. Following this procedure, a total of 10 environmental variables (Table S2) were ultimately selected for further analysis. These included six climatic variables, two topographic variables, one human activity variable, and one vegetation variable (Table 1). All selected variables, including HFI, showed low multicollinearity (VIF < 10; |r| < 0.7), ensuring their independent contributions. Based on the screening criterion that the cumulative contribution must exceed 85% [37,38], the key environmental variables influencing the species’ distribution were identified. Optimal habitat conditions were characterized by environmental variable ranges where the predicted presence probability was ≥0.5 [39].

### 2.3. Optimization and Prediction Using MaxEnt Model

#### 2.3.1. Parameter Optimization of MaxEnt Model

Uncalibrated species distribution models are prone to systematic bias in predicted probabilities, which may lead to significant discrepancies between model outputs and actual species distribution patterns [40]. If directly applied to predict the potential distribution of *P. tenuiflora*, such models could consequently result in overestimation or underestimation of suitable habitat areas. To improve predictive reliability, systematic parameter optimization was performed using the ENMeval package (v2.0.4) in R 4.2.2. Specifically, the ENMeval package (v2.0.4) was employed to tune the model parameters [41,42], with particular focus on two core parameters: the regularization multiplier (RM, tested in the range of 0.5 to 4.0 with a step size of 0.5) and the feature classes (FC). Based on five fundamental feature types (linear [L], quadratic [Q], product [P], threshold [T], and hinge [H]) [43], nine feature class combinations were constructed, which included L, H, LQ, HPT, LQH, QHP, QHPT, and LQHPT [44]. Subsequently, model selection was conducted using the corrected Akaike Information Criterion (AICc) based on the results from 10 replicated optimization runs, wherein the parameter set exhibiting the lowest average AICc value across these replicates was identified as optimal [45]. Spatial block cross-validation was employed within ENMeval to account for spatial autocorrelation. Finally, the best parameter set identified through this process was used to drive the final predictions, thereby significantly enhancing both the scientific validity and robustness of the projected potential distribution of *P. tenuiflora* under climate change scenarios. Delta.AICc was employed as the evaluation metric for assessing parameter optimization effectiveness of the MaxEnt model in this study [46]. The optimized model (RM = 3, FC = HPT) achieved the lowest AICc score among all tested parameter combinations, outperforming the default settings by ΔAICc > 2 [47], indicating substantially better performance while controlling model complexity.

#### 2.3.2. Parameter Configuration of the MaxEnt Model

The parameter-optimized MaxEnt model (RM = 3, FC = HPT) was employed in this study to predict the potential suitable habitats of *P. tenuiflora*. To enhance model accuracy and result robustness, the entire modeling process was conducted according to the following procedure: To account for spatial autocorrelation and obtain a realistic estimate of model performance, we implemented spatial block cross-validation. Using the R package blockCV version 3.0.1, we created five spatially independent folds, which were then used in MaxEnt for model training and testing. This approach ensures that model evaluation is based on predictions in geographically separate areas, mitigating inflated performance metrics. Subsequently, other key model parameters were configured in accordance with best practices in ecological niche modeling: the number of background points was set to 10,000 to adequately capture environmental variability. The model was run 10 times with different random seeds to reduce stochastic variability, and the results were averaged to produce the final ensemble output. Finally, the output of potential distribution was expressed as a logistic probability surface and saved in ASC raster format to facilitate subsequent spatial analysis and visualization.

#### 2.3.3. Evaluation of Optimized Model Prediction Accuracy

We further employed spatial block cross-validation using the ENMeval package to assess model transferability. The study area was partitioned into four spatially independent blocks based on longitude and latitude lines, each used once as a testing set while the remaining blocks were used for training. This approach helps to evaluate model performance under spatial independence and reduces inflated accuracy metrics due to spatial autocorrelation. In addition to the spatial cross-validation, three metrics, namely the area under the curve (AUC) of the receiver operating characteristic (ROC) curve, the true skill statistic (TSS), and Cohen’s kappa coefficient (Kappa), were selected in this study to systematically evaluate the predictive performance of the MaxEnt model. It is widely recognized that higher values of AUC, TSS, and Kappa generally indicate better predictive accuracy [48]. Specifically, the AUC value ranges from 0 to 1 [49], and its ecological interpretation is categorized as follows: 0.5–0.6 indicates failed prediction, 0.6–0.7 represents low accuracy, 0.7–0.8 denotes moderate accuracy, 0.8–0.9 reflects high accuracy, and 0.9–1.0 corresponds to very high accuracy [50]. Similarly, TSS is interpreted according to the following thresholds: 0.2–0.5 indicates poor model performance, 0.6–0.8 suggests good performance, and values above 0.8 represent excellent performance [51]. Likewise, the Kappa coefficient is evaluated based on established criteria: values below 0.4 are considered to indicate poor agreement, 0.4–0.75 indicates good agreement, and values above 0.75 represent excellent agreement [52].

### 2.4. Delineation of Potential Suitable Habitats

Based on the average results derived from 10 replicated cross-validations of the MaxEnt model (in ASC format), the data were uniformly converted to TIFF file with a spatial resolution of 2.5′ using ArcGIS 10.8 and were subsequently standardized to ensure both accuracy and compatibility in subsequent spatial analysis and mapping. The ‘Maximum Training Sensitivity plus Specificity’ logistic threshold was used to determine the binary classification of suitable habitats for *P. tenuiflora* [53]. Accordingly, the habitat suitability probability (P) was categorized into four consecutive grades: non-suitable area (0 ≤ P < 0.11), low-suitable area (0.11 ≤ P < 0.22), moderately suitable area (0.22 ≤ P < 0.5), and highly suitable area (0.5 ≤ P ≤ 1).

### 2.5. Spatial Pattern Changes in Potential Suitable Habitats

Under climate change, the spatial pattern of species suitable habitats is typically classified into three categories: retained areas, lost areas, and expanded areas [54,55,56]. To compare the dynamic changes in suitable habitats for *P. tenuiflora* across different periods and climate scenarios, the predicted suitability outputs from the MaxEnt model were systematically processed. Specifically, ASC files from each period were first converted to vector format using ArcGIS 10.8. A threshold value of 0.08 was then applied to binarize the presence probability of *P. tenuiflora*, thereby delineating suitable and non-suitable areas across China. Subsequently, the intersection tool in overlay analysis was employed to superimpose future and current suitable habitat vector data. The resulting overlapping areas were then converted back to raster format, which enabled both systematic identification of changes in suitable habitats and explicit delineation of retained, lost, and expanded zones within the potential suitable habitat distribution.

### 2.6. Centroid Shift in Potential Suitable Habitats

The centroid positions of suitable distribution areas for *P. tenuiflora* were determined, and their migration distances were estimated using SDMtoolbox 2.6 in this study [57]. Based on the suitability predictions generated by the MaxEnt model, continuous probability raster data were first converted into binary vector data, wherein areas with a distribution probability of *p* ≥ 0.08 were classified as suitable habitats, while those with *p* < 0.08 were considered non-suitable. Subsequently, the Band Collection Statistics tool in spatial analysis was applied, with the “Mean Center” selected as the geometric type, to extract the centroid coordinates of suitable areas under current climatic conditions as well as for the 2050s, 2070s, and 2090s under three climate scenarios: SSP126, SSP370, and SSP585. Finally, by comparing centroid shifts across different periods and scenarios, migration distances were further calculated to assess the potential impact of future climate change on the distribution center of *P. tenuiflora*.

## 3. Results

### 3.1. Calibration and Accuracy Evaluation of the Maxent Model

**Figure 2:**
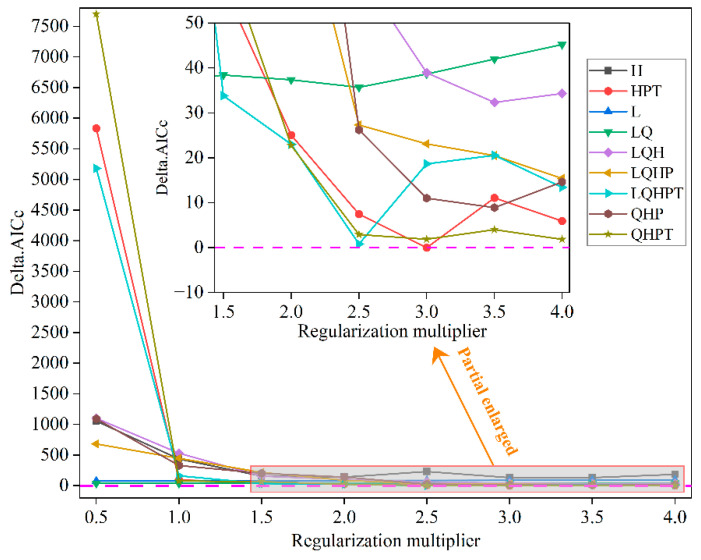
Delta.AICc derived from ENMeval-based model evaluation metrics for *P. tenuiflora*. Feature categories in legend: L (Linear), Q (Quadratic), H (Hinge), P (Product), T (Threshold). The short horizontal pink dashed line indicates the zero reference.

Initially, when default parameter settings (RM = 1, FC = LQHP) were applied, the model’s Delta.AICc value reached 446.639 (Figure 2), which significantly exceeded the threshold (Delta.AICc > 2). This result indicated substantial uncertainty in the default-parameter model and necessitated further optimization to improve predictive accuracy. Therefore, a systematic evaluation of different parameter combinations was conducted using the ENMeval package, incorporating 169 occurrence records of *P. tenuiflora* and 10 key environmental variables. Ultimately, an optimal parameter set satisfying the AICc information criterion was identified: RM = 3 and FC = HPT, with a Delta.AICc value of 0. This configuration demonstrated a significant enhancement in the model’s goodness-of-fit and overall reliability.

A systematic comparison and validation were conducted between the default parameter set (RM = 1, FC = LQHP) and the optimized parameter set (RM = 3, FC = HPT) for modeling performance in this study. Based on ensemble results from 10 replicated cross-validations, the AUC value under default parameters was determined to be 0.939 (Figure S1a), while that under optimized parameters was 0.930 (Figure S1b). Notably, both values were significantly higher than the “excellent” model discrimination threshold (AUC ≥ 0.9). Although a minor decrease in AUC was observed (ΔAUC = −0.009), a bootstrapped comparison of the ROC curves found no significant difference (*p* > 0.05), indicating that the optimized model retained statistically comparable discriminative power. This, combined with the significant improvement in model simplicity (ΔAICc = 0), demonstrates that overfitting issues inherent in the default settings were effectively mitigated through enhanced regularization (RM = 3) and a simplified feature class configuration (FC = HPT).

### 3.2. Key Environmental Variables Influencing the Distribution of P. tenuiflora

**Figure 3:**
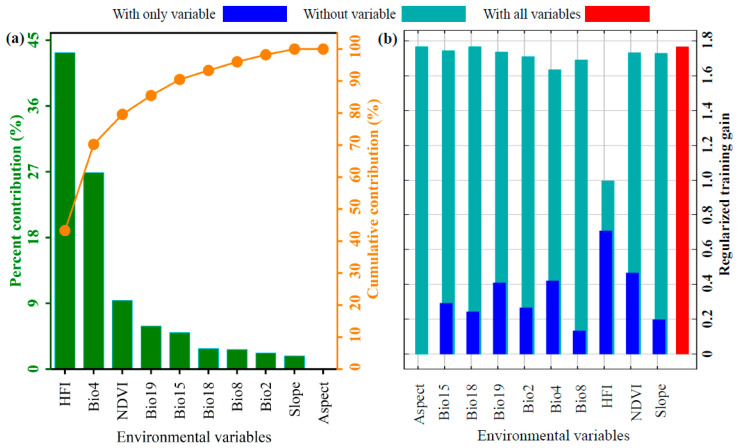
(**a**) Contribution and (**b**) jackknife test of regularized training gain for environmental variables in the MaxEnt model of *P. tenuiflora*.

The influence of 10 environmental variables on the potential geographical distribution of *P. tenuiflora* was evaluated using the optimized MaxEnt model, in combination with the contribution rate of environmental factors and the jackknife test. As illustrated in Figure 3a, the top five environmental factors affecting the distribution, listed in descending order of importance, were Human Footprint Index (HFI, 43.3%), Standard deviation of temperature seasonality (Bio4, 26.9%), Normalized Difference Vegetation Index (NDVI, 9.4%), precipitation of coldest quarter (Bio19, 5.9%), and variation in precipitation seasonality (Bio15, 5.0%). These factors were categorized into four groups: climate, human activities, vegetation, and topography, with cumulative contribution rates of 45.5%, 43.3%, 9.4%, and 1.8%, respectively. Furthermore, the jackknife test based on regularized training gain revealed that under the “with only variable” mode, the five factors yielding the highest gain values were HFI, NDVI, Bio4, Bio19, and Bio15 (Figure 3b). According to the screening criterion requiring a cumulative contribution exceeding 85%, HFI, Bio4, NDVI, and Bio19 were identified as the key environmental variables influencing the distribution of *P. tenuiflora*, with a cumulative contribution rate of 85.5%.

**Figure 4:**
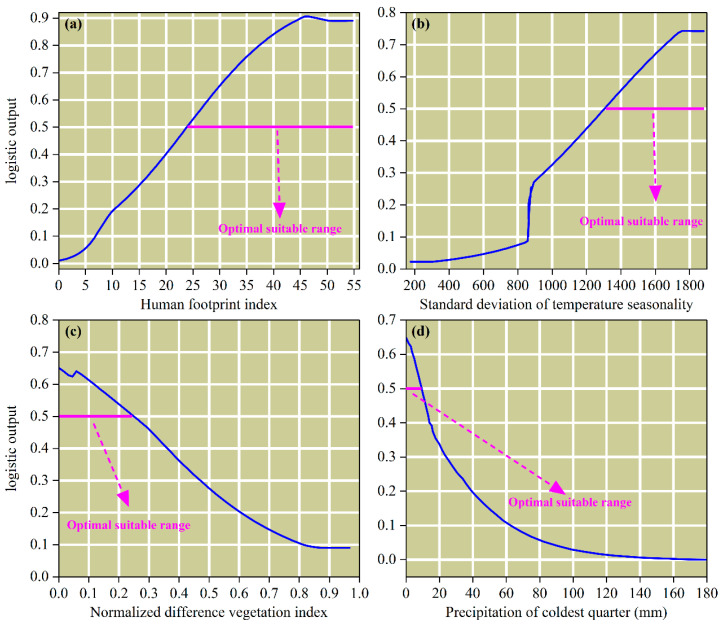
Response curves of major environmental variables in the distribution model of *P. tenuiflora*. (**a**) Human footprint index (HFI); (**b**) Standard deviation of temperature seasonality (Bio4); (**c**) Normalized difference vegetation index (NDVI); (**d**) Precipitation of coldest quarter (Bio19).

The response relationships between the presence probability of *P. tenuiflora* and four key environmental variables, namely HFI, Bio4, NDVI, and Bio19, were analyzed using single-factor response curves in this study (Figure 4). The results indicated that when HFI ranged from 0 to 46.53, the presence probability rapidly increased to a peak of 0.91, after which it slightly decreased and stabilized (Figure 4a). Similarly, as Bio4 increased from 175.78 to 1756.03, the presence probability exhibited multi-stage changes including slow growth, sharp increase, and rapid rise, followed by a slight decline beyond 1756.03 (Figure 4b). In comparison, when NDVI increased from 0 to 0.86, the presence probability decreased from 0.65 to 0.09 and subsequently remained stable (Figure 4c). Conversely, for Bio19, the presence probability dropped sharply within the range of 0 to 60 mm, then declined more gradually between 60 and 140 mm, and finally stabilized (Figure 4d). Based on the environmental variable ranges corresponding to a presence probability not less than 0.5, the optimal habitat conditions for *P. tenuiflora* were further determined (Figure 4). Specifically, these conditions were defined as follows: HFI between 24.41 and 55.00, Bio4 between 1303.36 and 1881.46, NDVI between 0 and 0.25, and Bio19 between 0 and 9.10 mm.

### 3.3. Potential Suitable Habitats of P. tenuiflora in China Under Current Climatic Conditions

**Figure 5:**
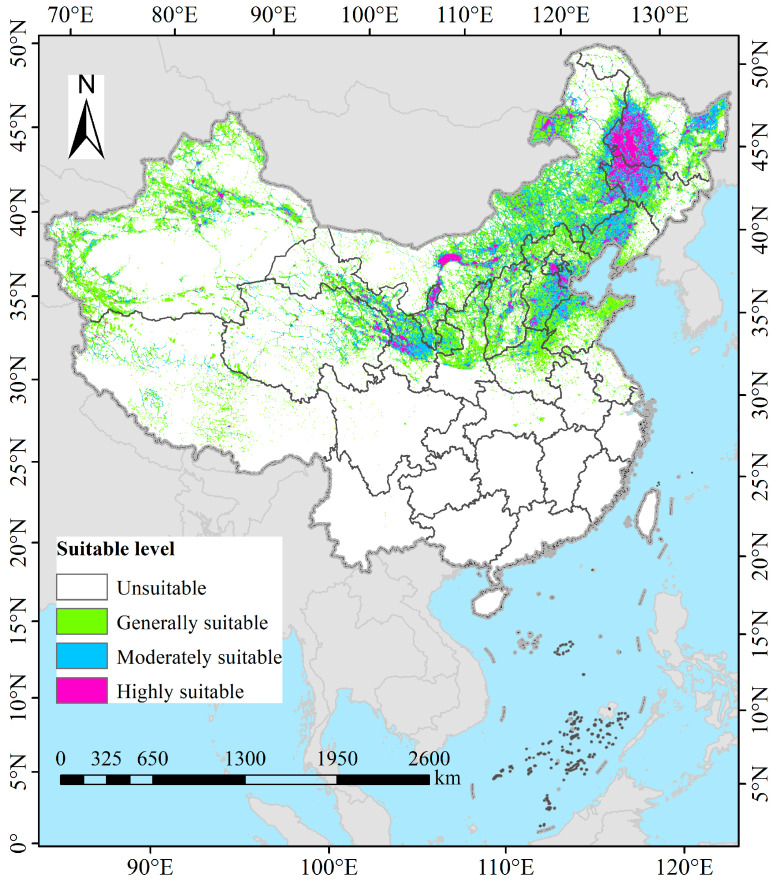
The potential geographic range of *P. tenuiflora* across China under current climate conditions.

Under current climatic conditions, the suitable habitats for *P. tenuiflora* were predominantly distributed in northern China (Figure 5). The total area of suitable habitats was estimated to be 258.26 × 10^4^ km^2^, accounting for approximately 26.90% of the total land area in China. Specifically, the highly suitable area covered about 27.36 × 10^4^ km^2^, representing 2.85% of the national territory, and was mainly concentrated in Heilongjiang and Jilin Provinces, with scattered distributions also observed in Inner Mongolia Autonomous Region, Liaoning Province, Beijing, and Tianjin. Furthermore, the moderately suitable area encompassed approximately 79.75 × 10^4^ km^2^, constituting 8.31% of the total land area in China. Its spatial distribution partially overlapped with the highly suitable habitats and extended into additional regions including Shandong Province, Hebei Province, Xinjiang Uygur Autonomous Region, and Qinghai Province. Finally, the low-suitable area amounted to 151.15 × 10^4^ km^2^, accounting for 15.75% of the total land area in China. This category was widely distributed across multiple northern provinces and extended southward into several southern regions, including Sichuan Province, Hubei Province, Anhui Province, and Jiangsu Province.

### 3.4. Potential Suitable Habitats of P. tenuiflora in China Under Future Climate Scenarios

**Figure 6:**
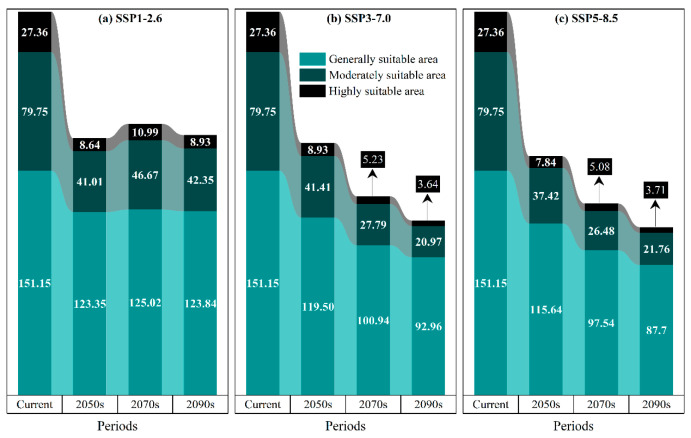
Projected changes (from the present to the 2090s) in the distribution of suitable habitat for *P. tenuiflora* under climate change scenarios (unit: 10^4^ km^2^).

**Figure 7:**
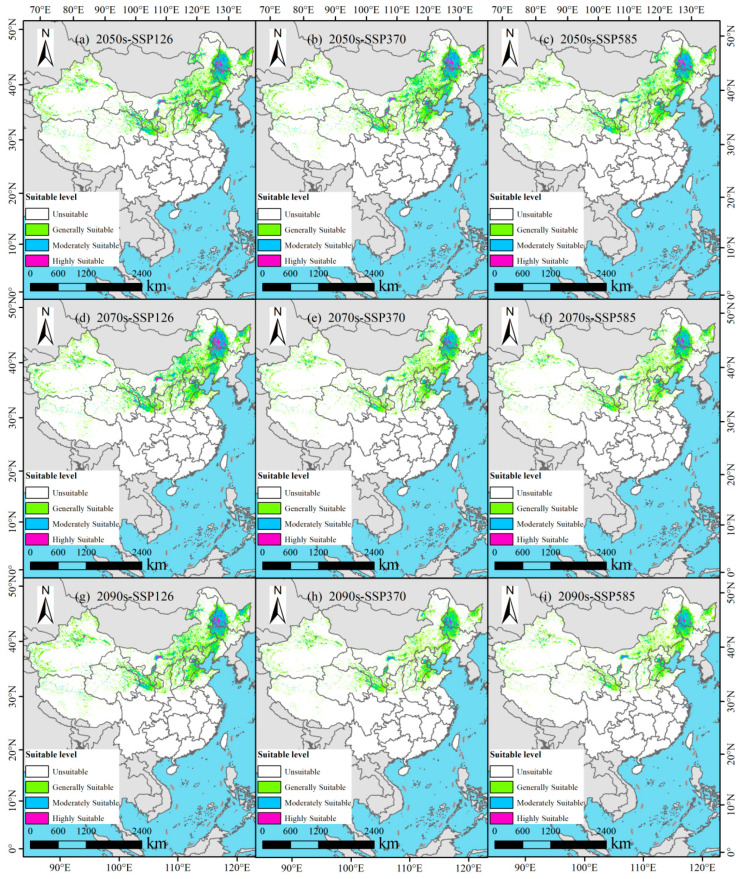
Projected distribution of suitable habitat for *P. tenuiflora* in China across multiple future climate scenarios.

Under the SSP126 scenario, the distribution of suitable habitats for *P. tenuiflora* exhibited notable spatiotemporal dynamics (Figure 6a and Figure 7). By the 2050s (Figure 7a), the total suitable area reached 173.00 × 10^4^ km^2^. This included a highly suitable area of 8.64 × 10^4^ km^2^ (approximately 0.90% of China’s total land area), predominantly located in Heilongjiang Province. The moderately suitable area covered 41.01 × 10^4^ km^2^ (4.27%), mainly distributed across Heilongjiang and Jilin Provinces. The low-suitable area accounted for 123.35 × 10^4^ km^2^ (12.85%), spread widely across the Inner Mongolia Autonomous Region, Liaoning Province, Hebei Province, Tianjin Municipality, Gansu Province, and Ningxia Hui Autonomous Region. By the 2070s (Figure 7d), the total suitable area increased to 182.68 × 10^4^ km^2^. The highly suitable area expanded to 10.99 × 10^4^ km^2^ (1.15%), still concentrated primarily in Heilongjiang. The moderately suitable area grew to 46.67 × 10^4^ km^2^ (4.86%), remaining largely within northeastern China. Meanwhile, the low-suitable area reached 125.02 × 10^4^ km^2^ (13.02%), with Shanghai Municipality emerging as a newly suitable region compared to the 2050s. By the 2090s (Figure 7g), the total suitable area experienced a slight decline to 175.11 × 10^4^ km^2^. Within this, the highly suitable area was recorded at 8.93 × 10^4^ km^2^ (0.93%), and the moderately suitable area measured 42.35 × 10^4^ km^2^ (4.41%), both still chiefly confined to Heilongjiang and Jilin Provinces. The low-suitable area covered 123.84 × 10^4^ km^2^ (12.90%), maintaining a spatial distribution consistent with that of the 2070s.

Under the SSP370 scenario, a continuous decline in the total area of suitable habitats was projected (Figure 6b and Figure 7). By the 2050s (Figure 7b), the total suitable area was estimated at 169.83 × 10^4^ km^2^. The highly suitable area accounted for 8.93 × 10^4^ km^2^ (0.93%), concentrated primarily in Heilongjiang Province. The moderately suitable area covered 41.41 × 10^4^ km^2^ (4.31%), exhibiting a spatial pattern consistent with that under the SSP126-2050s scenario but with a slightly larger extent. The low-suitable area amounted to 119.50 × 10^4^ km^2^ (12.45%), distributed across the Inner Mongolia Autonomous Region, Liaoning Province, Hebei Province, Tianjin Municipality, Gansu Province, and Ningxia Hui Autonomous Region. By the 2070s (Figure 7e), the total suitable area decreased to 133.86 × 10^4^ km^2^. Specifically, the highly suitable area diminished to 5.23 × 10^4^ km^2^ (0.55%), yet remained mainly within Heilongjiang Province. The moderately suitable area contracted to 27.79 × 10^4^ km^2^ (2.90%), becoming largely confined to Heilongjiang Province. Meanwhile, the low-suitable area was recorded at 100.94 × 10^4^ km^2^ (10.52%), still present in the same provincial-level regions as during the 2050s. By the 2090s (Figure 7h), the total suitable area further declined to 117.57 × 10^4^ km^2^. Notably, the highly suitable area was reduced to 3.64 × 10^4^ km^2^ (0.38%), showing a markedly contracted distribution within Heilongjiang Province. The moderately suitable area covered 20.97 × 10^4^ km^2^ (2.18%), continuing to concentrate predominantly in Heilongjiang Province. The low-suitable area accounted for 92.96 × 10^4^ km^2^ (9.68%), distributed mainly across the Inner Mongolia Autonomous Region, Liaoning Province, Hebei Province, Tianjin Municipality, Gansu Province, and Ningxia Hui Autonomous Region.

Under the SSP585 scenario, a continuous and pronounced reduction in suitable habitat area was projected for *P. tenuiflora* (Figure 6c and Figure 7). By the 2050s (Figure 7c), the total suitable area was estimated to be 160.90 × 10^4^ km^2^. The highly suitable area accounted for 7.84 × 10^4^ km^2^ (0.82%), located mainly within Heilongjiang Province. The moderately suitable area covered 37.42 × 10^4^ km^2^ (3.90%), concentrated primarily in Heilongjiang Province and Jilin Province. The low-suitable area reached 115.64 × 10^4^ km^2^ (12.05%), distributed widely across the Inner Mongolia Autonomous Region, Liaoning Province, Hebei Province, Tianjin Municipality, Gansu Province, and the Ningxia Hui Autonomous Region. By the 2070s (Figure 7f), the total suitable area decreased to 129.10 × 10^4^ km^2^. Specifically, the highly suitable area diminished to 5.08 × 10^4^ km^2^ (0.53%), remaining primarily within Heilongjiang Province. The moderately suitable area also declined, covering 26.48 × 10^4^ km^2^ (2.76%), and was distributed across parts of Heilongjiang Province and Jilin Province. Furthermore, the low-suitable area contracted to 97.54 × 10^4^ km^2^ (10.16%), while maintaining a similar spatial distribution pattern across the regions. By the 2090s (Figure 7i), the total suitable area further contracted to 113.16 × 10^4^ km^2^. Notably, the highly suitable area was reduced to only 3.71 × 10^4^ km^2^ (0.39%), and the moderately suitable area accounted for 21.76 × 10^4^ km^2^ (2.27%), with both classes confined mainly to Heilongjiang Province. Finally, the low-suitable area covered 87.70 × 10^4^ km^2^ (9.14%), predominantly distributed across the Inner Mongolia Autonomous Region, Liaoning Province, Hebei Province, Tianjin Municipality, Gansu Province, and the Ningxia Hui Autonomous Region.

### 3.5. Change Patterns of Suitable Habitats for P. tenuiflora Across Different Periods

**Figure 8:**
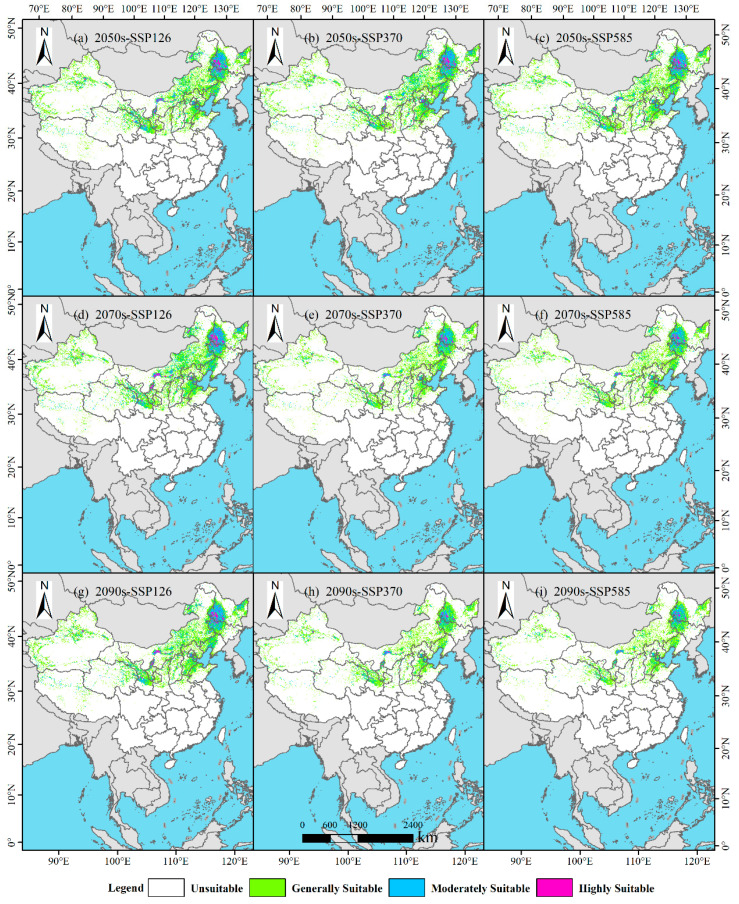
Spatiotemporal changes across stability, loss, and gain in the distribution of *P. tenuiflora*.

Under the SSP126 climate scenario, distinct phased characteristics were observed in the dynamic changes in suitable habitats for *P. tenuiflora* (Figure 8 and Table 2). Specifically, in the 2050s (Figure 8a), the retained area of suitable habitat was measured at 208.70 × 10^4^ km^2^, with a retention rate of 65.76%. Concurrently, the lost area was recorded as 106.35 × 10^4^ km^2^, accounting for 33.51% of the original habitat, while the expanded area remained relatively small at only 2.34 × 10^4^ km^2^, representing an expansion rate of 0.74%. By the 2070s (Figure 8d), the retained area increased to 220.94 × 10^4^ km^2^, and the retention rate rose to 69.71%. Simultaneously, the lost area decreased to 94.11 × 10^4^ km^2^, with a corresponding loss rate of 29.69%. Additionally, the expanded area slightly declined to 1.91 × 10^4^ km^2^, resulting in an expansion rate of 0.60%. In the 2090s (Figure 8g), the retained area decreased to 211.86 × 10^4^ km^2^, and the retention rate fell to 66.87%. Correspondingly, the lost area increased to 103.20 × 10^4^ km^2^, with a loss rate of 32.57%. Meanwhile, the expanded area further reduced to 1.77 × 10^4^ km^2^, representing an expansion rate of 0.56%. Overall, under this scenario, the suitable habitats were characterized by an initial increase followed by a decrease in retained area, an initial decrease followed by an increase in lost area, and a continuous slight decline in expanded area.

Under the SSP370 climate scenario, a continuous degradation trend was exhibited in the changes in suitable habitats (Figure 8 and Table 2). Specifically, in the 2050s (Figure 8b), the retained area was recorded as 206.24 × 10^4^ km^2^, with a retention rate of 65.27%. Meanwhile, the lost area reached 108.81 × 10^4^ km^2^, accounting for 34.44%, whereas the expanded area was only 0.94 × 10^4^ km^2^, with an expansion rate of 0.30%. By the 2070s (Figure 8e), the retained area significantly decreased to 163.10 × 10^4^ km^2^, and the retention rate dropped to 51.72%. Accordingly, the lost area increased to 151.95 × 10^4^ km^2^, with a loss rate of 48.18%. Furthermore, the expanded area further reduced to 0.31 × 10^4^ km^2^, representing an expansion rate of merely 0.10%. In the 2090s (Figure 8h), the retained area continued to decline to 142.65 × 10^4^ km^2^, and the retention rate decreased to 45.17%. Simultaneously, the lost area increased to 172.41 × 10^4^ km^2^, with a loss rate of 54.59%. However, the expanded area slightly recovered to 0.77 × 10^4^ km^2^, with an expansion rate of 0.24%. Overall, under this scenario, a continuous reduction in retained area and a continuous increase in lost area were observed, while the expanded area showed fluctuating characteristics, decreasing initially and then increasing.

Under the SSP585 climate scenario, a continuous and pronounced reduction trend was demonstrated in the changes in suitable habitats (Figure 8 and Table 2). Specifically, in the 2050s (Figure 8c), the retained area was measured at 195.19 × 10^4^ km^2^, with a retention rate of 61.74%. Meanwhile, the lost area reached 119.86 × 10^4^ km^2^, accounting for 37.91%, while the expanded area remained small at only 1.09 × 10^4^ km^2^, with an expansion rate of 0.35%. By the 2070s (Figure 8f), the retained area further decreased to 157.05 × 10^4^ km^2^, and the retention rate declined to 49.78%. Simultaneously, the lost area increased to 158.01 × 10^4^ km^2^, with a loss rate of 50.08%. Additionally, the expanded area reduced to 0.44 × 10^4^ km^2^, representing an expansion rate of only 0.14%. In the 2090s (Figure 8i), the retained area continued to shrink to 137.71 × 10^4^ km^2^, and the retention rate fell to 43.66%. In contrast, the lost area significantly increased to 177.34 × 10^4^ km^2^, with a loss rate as high as 56.23%. Meanwhile, the expanded area also decreased to 0.33 × 10^4^ km^2^, with an expansion rate of 0.11%. Overall, under the SSP585 scenario, a continuous reduction in retained area, a continuous increase in lost area, and a gradual decline in expanded area were observed.

### 3.6. Centroid Shifts in Suitable Habitats for P. tenuiflora Under Different Climate Scenarios

**Figure 9:**
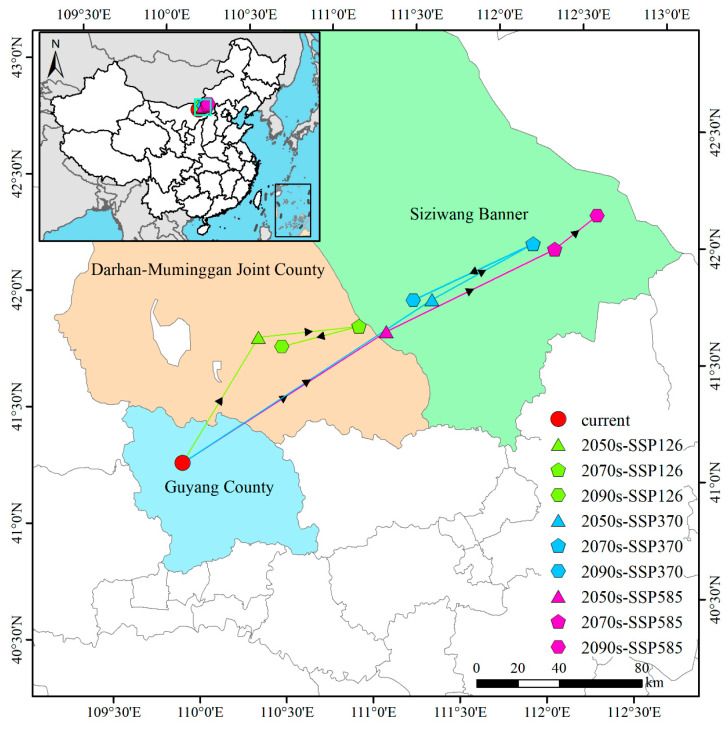
Migration of the geographic centroid of suitable habitat occupied by *P. tenuiflora* under climate change scenarios.

Under current climatic conditions, the centroid of suitable habitats for *P. tenuiflora* was in Xingshunxi Town, Guyang County, Baotou City, Inner Mongolia Autonomous Region (109°58′ E, 41°14′ N) (Figure 9). Reported centroid shifts are rounded to the nearest 10 km to better reflect the inherent uncertainty in species distribution projections under future climate scenarios. Under future climate scenarios, three distinct migration patterns of the centroid were identified: (1) Under the SSP126 scenario, the centroid exhibited an initial northeastward shift followed by a subsequent southwestward movement. In the 2050s, it was first displaced ~70 km northeast to Chaganhad Sumu, Darhan Mumingan Joint Banner (110°27′ E, 41°45′ N). Later, by the 2070s, it was further moved ~50 km northeast to Xisudertu (111°03′ E, 41°46′ N). Finally, in the 2090s, the centroid shifted ~39 km southwest, ultimately settling in Darhan Sumu, Darhan Mumingan Joint Banner (110°35′ E, 41°42′ N). (2) Under the SSP370 scenario, a more pronounced northeastward migration followed by partial retraction was observed. Initially, during the 2050s, the centroid was relocated ~145 km northeast to Hongor Sumu, Siziwang Banner, Ulanqab City (111°29′ E, 41°52′ N). Subsequently, by the 2070s, it advanced an additional ~56 km northeast to Harihada (112°06′ E, 42°05′ N). However, in the 2090s, the centroid retreated ~65 km southwest, ultimately reaching Qiqir, Siziwang Banner (111°23′ E, 41°52′ N). (3) Under the SSP585 scenario, a consistent northeastward migration pattern was maintained throughout the period. First, in the 2050s, the centroid was displaced ~118 km northeast to Xiaowengong Township, Siziwang Banner (111°12′ E, 41°45′ N). Then, by the 2070s, it progressed another ~92 km northeast to Gongjitang Town (112°14′ E, 42°03′ N). Ultimately, in the 2090s, the centroid advanced a further ~26 km northeast, finally reaching Baiyinchaoketu Town (112°30′ E, 42°11′ N). In summary, substantial spatial displacements of the centroid were observed across all climate scenarios. Whereas the SSP126 pathway demonstrated bidirectional fluctuation with eventual southwestern retraction, both the SSP370 and SSP585 pathways exhibited stronger and more persistent northeastward migration tendencies, reflecting differential responses to varying emission scenarios (Figure 9).

## 4. Discussion

### 4.1. Optimization and Validation of the MaxEnt Model for Predicting the Distribution of P. tenuiflora

While hybrid mechanistic models represent the cutting edge in species distribution modeling, optimized correlative approaches like ours remain widely applied and validated for projecting climate impacts, particularly for species with limited physiological data [53]. Our systematic parameter optimization aligns with best practices in the SDM literature to enhance model reliability. Following now-standard best practices in species distribution modeling, we performed parameter optimization to enhance prediction accuracy and ecological interpretability [58,59]. In this study, systematic tuning of the MaxEnt model was conducted using the ENMeval package. Specifically, the performance of different regularization multipliers (with RM values ranging from 0.5 to 4.0) and feature classes (including nine combinations such as L, H, LQ, and HPT) was thoroughly evaluated, ultimately leading to the identification of RM = 3 and FC = HPT as the optimal parameter configuration. This optimization process significantly reduced model complexity and overfitting risk [60]. Our optimized parameter set (RM = 3, FC = HPT) can be contextualized by comparing it with choices for other graminoids. For example, a study on *Elymus dahuricus*, a grass often found in stressful habitats, employed a less regularized model (RM = 1.5, FC = LQH) [61]. In contrast, research on the perennial grass Panicum virgatum utilized stronger regularization (RM = 4.0, FC = LQH) [62]. Our intermediate RM value and the use of threshold features (T) for *P. tenuiflora* likely reflect its specific ecological niche as a halophyte, which may be characterized by sharper physiological thresholds to environmental stressors like salinity and drought, a response that hinge (H) and threshold (T) features are well-suited to capture.

Notably, the Delta.AICc value decreased sharply from 446.639 under default parameters to 0, clearly indicating that the model achieved optimal parsimony while maintaining high predictive capability [63]. Furthermore, when compared to earlier studies such as those on *Leymus secalinus* and *Carex alatauensis*, where default parameters were directly applied [54,64], the systematic parameter optimization approach adopted in this study considerably enhanced model robustness. The optimized model demonstrated excellent performance on both training and testing datasets, achieving an AUC value of 0.930. Although this represents a slight decrease compared to the default parameters, the reduction in model complexity and concurrent improvement in generalization capacity are considered more ecologically meaningful [65]. Overall, this optimization strategy aligns well with current trends in ecological niche modeling, which increasingly emphasize balancing model complexity with predictive reliability rather than solely pursuing high prediction accuracy.

For model validation, a comprehensive multi-metric evaluation system was adopted in this study, which included the AUC, TSS, and Kappa coefficient to ensure both comprehensiveness and reliability of the assessment results [66]. The obtained AUC value of 0.930 indicated excellent discriminative ability of the model, while the TSS and Kappa values also reached levels considered good or higher, thereby demonstrating stable performance across different evaluation criteria. Compared to earlier studies that relied solely on the AUC metric, this multi-indicator validation approach provides a more comprehensive reflection of model performance and helps avoid potential bias in evaluation outcomes. Furthermore, spatial filtering at a 2.5′ resolution was applied to the occurrence data, which effectively reduced the impact of sampling bias on the prediction results [47]. Additionally, through rigorous environmental variable screening based on |r| < 0.8 and VIF < 10 thresholds, a total of 10 environmental variables were ultimately retained, thereby ensuring independence among variables and enhancing model stability [52]. As a result, this complete model optimization and validation workflow not only provides a solid methodological foundation for subsequent distribution predictions but also offers a replicable example for similar studies in the field.

### 4.2. Key Environmental Factors Influencing the Distribution of Suitable Habitats for P. tenuiflora

The MaxEnt model identified the Human Footprint Index (HFI) as the variable with the highest contribution to the distribution model of *P. tenuiflora*, with a contribution rate of 43.3%, significantly exceeding the contributions of traditionally dominant climatic factors. While this contrasts with studies of other grass species where climate predominates [67], it points to a potentially critical role of human-mediated habitat modification at a macro scale. The response curve suggests that the species reaches peak suitability at moderate HFI values; this correlation is ecologically consistent with the known tolerance of *P. tenuiflora* to low-to-moderate disturbance (e.g., light grazing that maintains open habitats [68], whereas high-intensity disturbance is detrimental [69]. However, we emphasize that this interpretation, while plausible, remains speculative in the absence of field-validated land-use intensity data directly linking specific human activities to population performance. Furthermore, temperature seasonality (Bio4, 26.9% contribution) was determined to be the second most important factor, indicating high sensitivity of the species to temperature fluctuations. Although this characteristic shows some similarity with findings from Ma and Sun’s study on Stipa purpurea [70], the notable difference in contribution ranking reveals significant ecological strategy differentiation among grass species. Finally, this differentiation may be attributed to the unique physiological adaptation mechanisms of *P. tenuiflora*, including its tolerance to saline-alkali stress and specific response strategies to temperature variations [71,72,73]. These specialized traits potentially enable competitive advantage under specific environmental conditions, highlighting the species’ distinct niche requirements. The dominant role of HFI contrasts sharply with related grasses like *Leymus secalinus* where climate dominates [54], highlighting *P. tenuiflora*’s unique adaptation to human-disturbed saline environments. Similarly, its negative NDVI response differs from the positive correlation observed in most grassland species, reinforcing its specialization as a heliophilic halophyte.

The shape of the response curves provides deep ecological insights into the niche requirements of *P. tenuiflora*. First, the response curve for HFI, peaking between values of 24 and 55, aligns with the intermediate disturbance hypothesis [74,75]. This suggests that *P. tenuiflora* reaches its optimal abundance in moderately disturbed habitats (e.g., light grazing), which maintain open canopies and reduce competition from other grasses [68], whereas intact vegetation or intense human pressure create suboptimal conditions. Second, the significant negative response to NDVI underscores its specialization as a heliophilic halophyte and pioneer species. This pattern contrasts sharply with typical grassland species, which generally show increased suitability with higher NDVI [76], highlighting its unique adaptation to open, saline habitats with sparse vegetation and low competition. Finally, the sharp decline in suitability with increasing Bio19 (winter precipitation) is consistent with known stressors for halophytes. Increased cold-season rainfall may lead to salt leaching, reducing soil salinity below optimal levels [4], and/or cause waterlogging stress, leading to root anoxia [77]. Together, these responses illustrate how multiple environmental factors interact to shape the species’ distribution and highlight the need to incorporate edaphic variables in future fine-scale studies.

### 4.3. Change Trends in Suitable Habitats of P. tenuiflora Under Climate Change Scenarios

Projections under multiple climate scenarios (SSP126, SSP370, SSP585) in this study indicate that the suitable habitat area of *P. tenuiflora* is expected to undergo significant reduction, accompanied by fundamental changes in spatial distribution patterns. Specifically, under the high-emission scenario (SSP585), the suitable area is projected to decrease to 113.16 × 10^4^ km^2^ by the 2090s, representing a 56.2% reduction compared to the current distribution. Notably, this magnitude of loss is substantially higher than predictions by Sun et al. [24] for climate suitability of natural forage grasses in Qinghai Province (approximately 30%), suggesting that *P. tenuiflora*, as a specialized halophytic grass species, may be highly vulnerable to climate change. This projected vulnerability is consistent with its narrow ecological niche, as defined by our model, which is characterized by specific thresholds of temperature seasonality (Bio4) and winter precipitation (Bio19). Species with such specialized requirements are theoretically more susceptible to niche displacement under rapid climate change [78,79]. Our macro-scale projection aligns with a growing body of literature highlighting the climate change risks to specialist species and saline ecosystems [80]. For instance, a recent study on *Elymus dahuricus* also reported significant range contractions under high-emission scenarios [61]. However, we acknowledge that correlative SDMs like ours cannot directly incorporate physiological mechanisms. The claim of ‘high sensitivity’ is inferred from the projected habitat loss and is supported by the known physiology of *P. tenuiflora* from independent, controlled studies, which detail its specific thresholds for salt tolerance [14] and water-use efficiency [23]. Ultimately, integrating these physiological thresholds into a mechanistic model represents a critical next step to move beyond our correlative findings and validate the projected sensitivity.

To assess the ecological plausibility of these projected changes, we compared our findings with those for other typical grassland species in Northern China. For instance, studies projected habitat losses of approximately 30–40% by the 2090s for *Stipa breviflora* and *Leymus chinensis* under high-emission scenarios [25,28]. The more substantial habitat contraction predicted for *P. tenuiflora* (up to 56.2% under SSP585) underscores its heightened vulnerability as a specialist halophyte. This greater sensitivity is ecologically plausible, as its narrow niche, defined by specific soil salinity and moisture regimes, is profoundly impacted by the synergistic effects of climate change and human activities (HFI).

Centroid shift analysis revealed distinctive spatial reorganization in the distribution pattern of *P. tenuiflora*. Under current climatic conditions, the centroid was in Guyang County, Baotou City, Inner Mongolia Autonomous Region (109°58′ E, 41°14′ N), whereas future climate scenarios consistently projected northeastward migration. Across both SSP370 and SSP585 scenarios, continuous northeastward displacement was observed, reaching a maximum shift of ~145 km toward central and northeastern Inner Mongolia. This pattern contrasted significantly with the altitudinal shifts reported for species such as *Elymus nutans* and *Elymus dahuricus*, which typically migrate to higher elevations [61,67], indicating divergent climate adaptation strategies. Under the SSP126 scenario, however, a more complex fluctuating trajectory was identified, featuring initial northeastward movement followed by southwestward retraction. This pattern suggests greater instability in species distribution under low-emission scenarios [61,62]. The observed migrations represent geographic displacements driven by climatic suitability tracking, yet these movements are constrained by multiple factors including topographic barriers, limited dispersal capacity, and fragmented habitat connectivity [81,82]. While models project northeastward centroid shifts under climate change, these represent potential climatic suitability shifts rather than predictions of actual population dispersal. Realized range shifts will likely be constrained by limited seed dispersal capacity, habitat fragmentation, and landscape barriers including the Greater Khingan Mountains, Yellow River basin, and extensive agricultural/urban areas. These factors may create significant lags between climate-driven habitat changes and actual species redistribution. Therefore, while our centroid analysis identifies the direction and magnitude of climatic pressure, future work integrating landscape connectivity models, such as circuit theory or least-cost path analysis, with our suitability projections is essential to map potential dispersal corridors and identify populations most at risk of being stranded in deteriorating climates.

### 4.4. Limitations of This Study and Future Research Directions

Five main limitations were identified in this study on climate change impacts on species distribution. First, as a correlative model, our approach inherently relies on historical climate-distribution relationships and does not incorporate key biological processes such as physiological tolerance, phenotypic plasticity, adaptive evolution, or species interactions (e.g., competition with invasive plants). Second, although we included multiple environmental dimensions, a critical omission was the lack of soil salinity data, a primary factor known to directly limit the distribution of halophytes like *P. tenuiflora*. Third, our projections are based on a single climate model (BCC-CSM2-MR). The absence of a multi-model ensemble from CMIP6 means we cannot quantify the uncertainty inherent in climate projections themselves, resulting in deterministic rather than probabilistic forecasts. Fourth, our methodology lacks cross-scale integration, failing to effectively link micro-scale mechanisms (e.g., gene expression, phenotypic adaptation) with macro-scale ecological dynamics (e.g., population migration, range shifts). Fifth, the static, correlative framework does not account for dynamic ecological processes such as dispersal limitation, population dynamics, and biotic interactions, which collectively shape realized distributions. To address these limitations, future research should: (1) develop process-based or hybrid models that integrate physiological thresholds and competition; (2) incorporate direct measurements of soil salinity and other edaphic factors; (3) employ CMIP6 multi-model ensembles to quantify prediction uncertainty; (4) establish cross-scale frameworks coupling environmental genomics with individual-based models; and (5) prioritize the collection of field data on demography and species interactions to validate and refine model projections.

Our projections of habitat change for *P. tenuiflora* have broader implications for associated biodiversity. As a foundational halophyte and a key species used in restoration, its presence typically exerts net positive effects on local flora and fauna. By improving soil structure, reducing surface salinity, and increasing organic matter, *P. tenuiflora* facilitates the colonization and growth of other, less tolerant plant species, acting as an ecosystem engineer. This facilitation effect can enhance plant diversity and productivity in saline-alkali grasslands. Consequently, the projected large-scale habitat loss for *P. tenuiflora* could trigger a cascade of negative impacts, potentially leading to reduced habitat complexity, lower soil fertility, and a decline in the diversity of dependent invertebrates, soil microbes, and other fauna. Therefore, conserving and restoring *P. tenuiflora* is critical not only for the species itself but also for maintaining the biodiversity and ecosystem function of the entire saline-alkali grassland community.

## 5. Conclusions

Through systematic optimization of the MaxEnt model and integration of multi-source environmental data, this study revealed the potential suitable habitat distribution pattern of *P. tenuiflora* in China and its response mechanisms to future climate change. Four key environmental factors were identified as major determinants of its distribution, human activity intensity HFI, temperature seasonality Bio4, vegetation index NDVI, and precipitation of the coldest quarter Bio19, collectively accounting for 85.5% of the explained variation. Particularly notable was the dominant role of HFI, underscoring the substantial influence of human disturbance on the distribution of this halophytic grass species. Under current climatic conditions, suitable habitats for *P. tenuiflora* were primarily concentrated in northern China, covering a total area of approximately 258.26 × 10^4^ km^2^. Under future climate scenarios SSP126, SSP370, and SSP585, habitat suitability was projected to decline, with losses intensifying as greenhouse gas concentrations increased. Under the high emission scenario SSP585, a reduction of 56.2% was predicted by the 2090s. Spatial analysis also indicated a northeastward shift in the centroid of suitable habitats, with a maximum displacement of 145.36 km, reflecting geographic relocation likely driven by climate warming. Beyond providing spatially explicit predictions of current and future distributions, this study highlighted the critical importance of multi factor interactions and parameter optimization in improving the robustness of species distribution models. Moreover, it established a scientific foundation for conservation planning and adaptive management of *P. tenuiflora*. To advance predictive capability, future studies should prioritize the integration of physiological mechanisms and multi-scale dynamic processes to enhance both mechanistic realism and practical applicability.

## Figures and Tables

**Table 1:** Ten environmental factors predicting potential distribution of *P. tenuiflora* in this study.

Category	Abbreviation	Environmental Variables	Units	VIF
Bioclimatic	Bio2	Mean diurnal range (Mean of monthly)	°C	2.63
	Bio4	Standard deviation of temperature seasonality		7.04
	Bio8	Mean temperature of wettest quarter	°C	7.13
	Bio15	Variation in precipitation seasonality		2.93
	Bio18	Precipitation of warmest quarter	mm	3.71
	Bio19	Precipitation of coldest quarter	mm	2.20
Topographic	Aspect	Aspect	°	3.31
	Slope	Slope	°	1.26
Human	HFI	Human footprint index		1.50
Vegetation	NDVI	Normalized difference vegetation index		1.57

**Table 2:** Shifts in the suitable habitat area of *P. tenuiflora* under differing projection scenarios.

Period	Area (10^4^ km^2^)			Rate of Change (%)		
	Stability	Contraction	Expansion	Stability	Contraction	Expansion
2050s-SSP126	208.70	106.35	2.34	65.76	33.51	0.74
2070s-SSP126	220.94	94.11	1.91	69.71	29.69	0.60
2090s-SSP126	211.86	103.20	1.77	66.87	32.57	0.56
2050s-SSP370	206.24	108.81	0.94	65.27	34.44	0.30
2070s-SSP370	163.10	151.95	0.31	51.72	48.18	0.10
2090s-SSP370	142.65	172.41	0.77	45.17	54.59	0.24
2050s-SSP585	195.19	119.86	1.09	61.74	37.91	0.35
2070s-SSP585	157.05	158.01	0.44	49.78	50.08	0.14
2090s-SSP585	137.71	177.34	0.33	43.66	56.23	0.11

## Data Availability

The original contributions presented in this study are included in this article. Further inquiries can be directed to the corresponding author.

## Supplementary Materials

The following supporting information can be downloaded at: https://www.mdpi.com/article/10.3390/biology14101426/s1, Table S1. Twenty-four environmental variables used in this study; Table S2. Spearman relationship coefficients and VIF of ten environmental factors; Figure S1. ROC-based accuracy assessment of the MaxEnt model for P. tenuiflora: (a) AUC under default parameter settings; (b) AUC under optimized parameter settings.
