# Supplementary material for: Potential Distribution and Response to Climate Change in Puccinellia tenuiflora in China Projected Using Optimized MaxEnt Model
Source: Biology (Basel). 2025 Oct 16;14(10):1426. doi: 10.3390/biology14101426 (PMC12561134; doi:10.3390/biology14101426)
Supplement: Supplementary file 1 [file biology-14-01426-s001.zip › biology-3901352-supplementary.pdf]

Table S1. Twenty-four environmental variables used in this study.

Category	Abbreviation	Environmental variables	Units	Range
Bioclimatic	Bio1	Annual mean temperature	°C	-9.55～14.75
	Bio2	Mean diurnal range (Mean of monthly)	°C	8.48～15.70
	Bio3	Isothermality (Bio2/Bio7) (× 100)		20.50～39.48
	Bio4	Standard deviation of temperature seasonality		850.08～1712.53
	Bio5	Max temperature of warmest month	°C	10.56～34.01
	Bio6	Min temperature of coldest month	°C	-33.98～-2.55
	Bio7	Temperature annual range (Bio5-Bio6)	°C	32.47～58.51
	Bio8	Mean temperature of wettest quarter	°C	2.19～26.31
	Bio9	Mean temperature of driest quarter	°C	-22.15～12.28
	Bio10	Mean temperature of warmest quarter	°C	2.19～26.49
	Bio11	Mean temperature of coldest quarter	°C	-25.25～2.68
	Bio12	Annual precipitation	mm	33.00～1009.00
	Bio13	Precipitation of wettest month	mm	7.00～258.00
	Bio14	Precipitation of driest month	mm	0.00～21.00
	Bio15	Variation of precipitation seasonality		34.16～139.67
	Bio16	Precipitation of wettest quarter	mm	15.00～555.00
	Bio17	Precipitation of driest quarter	mm	0.00～80.00
	Bio18	Precipitation of warmest quarter	mm	15.00～551.00
	Bio19	Precipitation of coldest quarter	mm	0.00～80.00
Topographic	Altitude	Altitude	m	1.00～5395.00
	Aspect	Aspect	°	0.00～5.32
	Slope	Slope	°	0.00～359.88
Human	HFI	Human footprint index		0.00～50.00
Vegetation	NDVI	Normalized difference vegetation index		0.05～0.54

Table S2. Spearman relationship coefficients and VIF of ten environmental factors.

r	bio2	bio4	bio8	bio15	bio18	bio19	slope	aspect	HFI	NDVI	VIF
bio2	1.0000	-0.2879	-0.6268	-0.3051	-0.6859	-0.4197	0.5157	0.0123	-0.3171	-0.4903	2.6251
bio4		1.0000	0.2630	0.5700	0.3283	0.0355	-0.5050	0.2519	0.2142	0.3067	7.0420
bio8			1.0000	0.4376	0.6150	0.0869	-0.6991	-0.1932	0.4104	0.3828	7.1338
bio15				1.0000	0.5299	-0.2431	-0.6330	0.1386	0.1803	0.3456	2.9258
bio18					1.0000	0.3566	-0.5862	0.0987	0.3542	0.5570	3.7116
bio19						1.0000	0.0419	0.0985	0.2966	0.1627	2.1954
slope							1.0000	0.0009	-0.3019	-0.4118	3.3142
aspect								1.0000	-0.0622	0.0613	1.2593
HFI									1.0000	0.2020	1.4984
NDVI										1.0000	1.5650

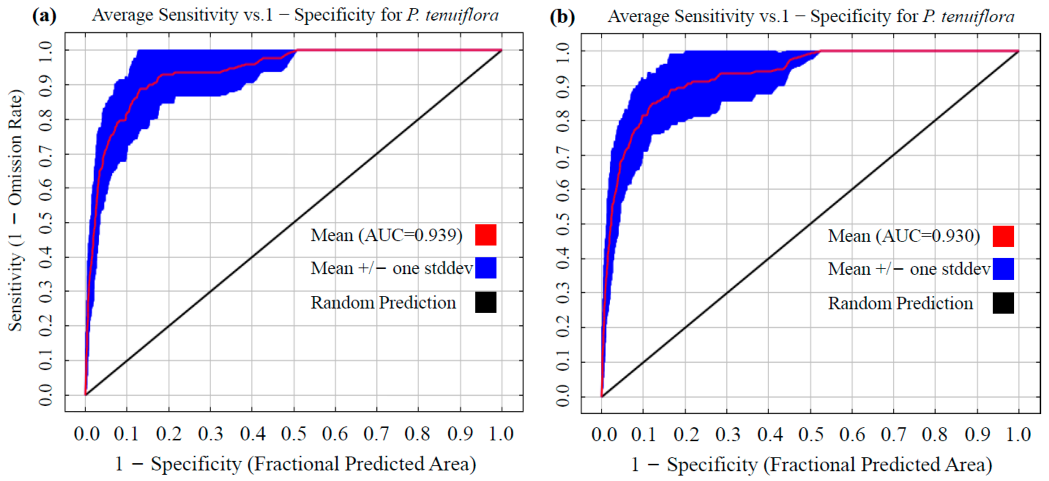

Figure S1. ROC-based accuracy assessment of the MaxEnt model for P. tenuiflora: (a) AUC under default parameter settings; (b) AUC under optimized parameter settings.
